# The FRAIL scale independently predicts 28-day mortality in critically ill older adults: a prospective comparison with the Clinical Frailty Scale

**DOI:** 10.3389/fmed.2026.1861664

**Published:** 2026-07-01

**Authors:** Ferhan Demirer Aydemir, Pinar Soysal, Nuri Mehmet Yakar, Bilgin Comert, Necati Ali Gokmen

**Affiliations:** 1 Department of Internal Medicine, Division of Intensive Care Medicine, Faculty of Medicine, Çanakkale Onsekiz Mart University, Çanakkale, Türkiye; 2 Department of Geriatrics, Faculty of Medicine, Bezmialem Vakif University, İstanbul, Türkiye; 3 Department of Internal Medicine, Division of Intensive Care Medicine, Faculty of Medicine, Dokuz Eylul University, Izmir, Türkiye

**Keywords:** Clinical Frailty Scale, critical illness, FRAIL scale, frailty, geriatric patients, intensive care unit, mortality prediction

## Abstract

**Background:**

Frailty is a multidimensional syndrome associated with adverse outcomes in older adults. This study aimed to compare the predictive value of the FRAIL scale and the Clinical Frailty Scale (CFS) for 28-day mortality among critically ill geriatric patients.

**Methods:**

In this prospective cohort study conducted in a tertiary intensive care unit, all consecutive patients aged ≥65 years were assessed for frailty at intensive care unit admission using the FRAIL scale and the CFS. Logistic regression analysis was performed to identify independent associations with 28-day mortality. Receiver operating characteristic analyses with DeLong comparison and Kaplan–Meier survival analyses with log-rank testing were also performed.

**Results:**

Among 168 patients, 53% were male, and the median age was 77 years (IQR 70–82). The 28-day mortality rate was significantly higher in frail patients compared with non-frail patients according to both the FRAIL scale (36% vs. 16%, *p* = 0.005) and CFS (34% vs. 15%, *p* = 0.009). Agreement between the two tools was good (*κ* = 0.75; 95% CI, 0.65–0.85). In multivariable analysis, frailty defined by the FRAIL scale remained independently associated with 28-day mortality (OR 2.41; 95% CI, 1.01–5.76; *p* = 0.048), whereas CFS-based frailty was not (OR 2.26; 95% CI, 0.91–5.63; *p* = 0.080). The AUROC values for total scores were 0.664 for the FRAIL scale and 0.700 for the CFS (DeLong *p* = 0.149); for dichotomized frailty status, AUROC values were 0.620 and 0.606, respectively (DeLong *p* = 0.595).

**Conclusion:**

Frailty at ICU admission is an important predictor of short-term mortality in older adults. The FRAIL scale demonstrated independent prognostic value in the primary adjusted model, while AUROC comparison did not show a statistically significant discrimination advantage over the CFS.

## 1 Introduction

Frailty is a multidimensional syndrome defined by a decline in physical, physiological, and cognitive reserves, leading to increased vulnerability to stressors and adverse outcomes (1). Frail individuals often present with reduced mobility, muscle weakness, weight loss, poor nutritional status, and cognitive impairment, which together limit their ability to recover from acute illness. Contemporary literature emphasizes that frailty becomes more common with age but remains distinct from chronological ageing, disability, and comorbidity (2–4). Rather than age itself, frailty reflects the accumulation of health deficits and loss of physiological resilience.

Frailty is also highly prevalent among patients admitted to intensive care units (ICUs) and has been consistently associated with longer hospital stays, increased morbidity, and higher short-term mortality (5). Assessing frailty at ICU admission provides important prognostic information about baseline health status and recovery potential. However, despite its clinical importance, there is no single accepted method for measuring frailty. Different tools capture different domains, and their performance can vary across settings (6). Choosing a tool that is both practical and predictive in the ICU remains a clinical and research challenge.

The Clinical Frailty Scale (CFS) is widely used because of its simplicity and bedside applicability. Several studies have shown that higher CFS scores are associated with increased mortality in older ICU patients (7–10). However, its predictive strength may weaken after adjusting for illness severity and comorbidities (11, 12). The FRAIL scale, which includes questions about fatigue, resistance, ambulation, illness, and weight loss, captures functional and nutritional aspects of frailty that may be particularly relevant in critical illness (13). Despite this, it has been less studied in ICU populations, and direct comparisons between the two scales are limited.

This prospective cohort study compared the predictive performance of the FRAIL scale and the Clinical Frailty Scale (CFS) for 28-day mortality among critically ill older adults. We hypothesised that both tools would be associated with short-term mortality and evaluated whether either instrument provided additional independent or discriminative prognostic information. As a secondary objective, we evaluated the agreement between the two instruments. To our knowledge, this is one of the first prospective studies to directly compare these frailty tools in a critically ill geriatric population, addressing an important gap in the current literature.

## 2 Methods

### 2.1 Study design and ethical approval

This study was approved by the Institutional Review Board (approval number 2020/30-19, dated 21 December 2020). It was designed as a prospective cohort study conducted in the intensive care unit (ICU) of Dokuz Eylul University Hospital (DEUH), a tertiary university hospital. Written informed consent was obtained from all participants or their legal representatives prior to inclusion.

### 2.2 Patients

**Figure 1:**
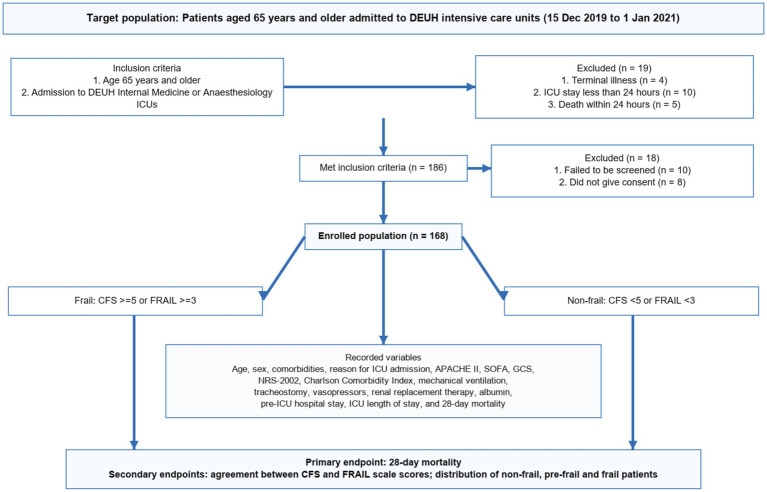
Study flow diagram showing the inclusion and exclusion process of patients assessed for frailty in the intensive care unit.

All consecutive patients aged 65 years or older who were admitted to the Internal Medicine and Anaesthesiology ICUs between 15 December 2019 and 1 January 2021 were screened for inclusion. For patients admitted to the ICU more than once during the study period, only the first admission was analysed. The patient selection process is summarized in Figure 1.

Patients with terminal illnesses, such as end-stage cancer or heart failure, were excluded. We also excluded those discharged alive within 24 h and those who died within 24 h, because frailty assessment and short-term outcome attribution may be less reliable in very early discharge or death. Patients with a confirmed diagnosis of COVID-19 were not included.

### 2.3 Data collection

Frailty status on ICU admission was the primary exposure variable. It was assessed by two members of the research team through interviews with patients and their relatives, and by reviewing medical records. Frailty assessments were performed by trained investigators who were not involved in outcome adjudication. Both the CFS and FRAIL scale were completed and documented on a standardised study form attached to each patient’s ICU chart.

The FRAIL scale comprises five components: (1) fatigue within the past 4 weeks, (2) difficulty climbing one flight of stairs, (3) difficulty walking more than 100 metres, (4) presence of five or more chronic illnesses, and (5) unintentional weight loss greater than 5% in the past year.

Each positive response scores one point, yielding a total score from 0 to 5. Patients scoring 0 were classified as non-frail, 1–2 as pre-frail, and ≥3 as frail (13). In the primary analysis, pre-frail and non-frail patients were combined because of the modest sample size and limited number of mortality events.

The CFS is a 9-point ordinal measure based on functional capacity and dependency, ranging from 1 (very fit) to 9 (terminally ill). A score greater than 4 was considered indicative of frailty on the index ICU admission day (4).

### 2.4 Clinical variables

Demographic and clinical data were recorded, including age, sex, comorbidities, reason for ICU admission (e.g., respiratory failure, shock, sepsis, trauma, cerebrovascular event, postoperative state), Acute Physiology and Chronic Health Evaluation II (APACHE II) score (14), Charlson Comorbidity Index (15), Sequential Organ Failure Assessment (SOFA) score (16), Glasgow Coma Score (GCS) (17), and Nutritional Risk Screening 2002 (NRS-2002) score (18).

Polypharmacy was defined as the use of five or more regular medications at the time of ICU admission (19, 20). Laboratory data, including serum albumin level, and the duration of hospitalisation prior to ICU transfer were also documented. We further recorded use and duration of mechanical ventilation, tracheostomy, vasopressors/vazoactive drugs, and renal replacement therapy.

### 2.5 Outcomes

The primary outcome was all-cause mortality within 28 days following ICU admission. For patients discharged before day 28, mortality data were verified using the national electronic death notification system.

The secondary outcome was the level of agreement between the CFS and FRAIL scores in the study cohort.

### 2.6 Statistical analysis

Continuous variables were expressed as mean ± standard deviation (SD) or median (interquartile range, IQR), as appropriate, and categorical variables as counts and percentages. Group comparisons were performed using the independent-samples *t*-test or the Mann–Whitney *U* test for continuous variables, and the Chi-square or Fisher’s exact test for categorical variables.

To examine the association between frailty—defined by either the FRAIL or CFS—and 28-day mortality, two separate multivariable logistic regression models were constructed. The FRAIL and CFS scores were entered as the primary independent variables. Clinically relevant covariates were included in the models, comprising age, sex, Charlson Comorbidity Index, APACHE II score, Glasgow Coma Score, NRS-2002, body mass index (BMI), presence of shock, cerebrovascular disease, and polypharmacy. Multicollinearity among covariates was assessed using variance inflation factors before inclusion in the multivariable models; all VIF values were below 3.0 (maximum VIF 2.76). Variables included in each multivariable model were selected based on clinical relevance and statistical significance in univariate analysis, while avoiding overfitting. Because comorbidity may act both as a confounder and as a component of frailty biology, CCI-adjusted models were considered conservative primary models, and sensitivity analyses excluding CCI were also performed. Crude and adjusted odds ratios (ORs) with corresponding 95% confidence intervals (CIs) were reported. Agreement between the FRAIL and CFS scales was evaluated using Cohen’s kappa statistic, with interpretation based on established benchmarks (21). AUROC analysis was used to evaluate discrimination for 28-day mortality, and paired AUROCs were compared using DeLong’s test. Kaplan–Meier curves and log-rank tests were used to compare survival according to frailty status.

All statistical analyses were performed using SPSS software version 23.0 (IBM Corp., Armonk, NY, USA). A two-tailed *p*-value <0.05 was considered statistically significant.

## 3 Results

### 3.1 Patient characteristics

A total of 168 patients were included in the analysis, of whom 53% were male (Figure 1). The median age was 77 years (IQR 70–82). Before ICU admission, most patients lived at home (93.5%), followed by elderly care centres (4.8%), nursing homes (1.2%), and palliative care facilities (0.6%). The primary reasons for ICU admission included acute respiratory failure (89.9%), shock (45.2%), postoperative status (25.6%), sepsis (12.5%), cerebrovascular disease (5.4%), trauma (1.2%), and head injury (0.6%), with some patients presenting with more than one indication (Table 1).

**Table 1:** Baseline characteristics of the study population.

Characteristic	FRAIL scale and Clinical Frailty Scale (CFS)						Total (*n* = 168)
	Frail FRAIL ≥3 *n* = 95	Not frail FRAIL <3 *n* = 73	*p*	Frail CFS ≥ 5 *n* = 113	Not frail CFS < 5 *n* = 55	*p*	
Male, *n* (%)	48 (50.5)	41 (56)	0.46	58 (51)	31(56.4)	0.53	89 (53)
Age, Median (25–75%)	78 (72–85)	73 (68–80)	**0.001**	79 (72–85)	71 (67–78)	**<0.001**	77 (70–82)
Place of acceptance, *n* (%)							
House	88 (92.6)	69 (94)	0.31	105 (93)	52 (94.5)	0.48	157 (93.5)
Resting home	4 (4.2)	4 (5)		5 (4)	3 (5.5)		8 (4.8)
Nursing home	2 (2.1)	0 (0)		2 (1.8)	0 (0)		2 (1.2)
Palliative center	1 (1.1)	0 (0)		1 (0.9)	0 (0)		1 (0.6)
Diagnosis on admission, *n* (%)							
Respiratory failure	85 (89.5)	66 (90)	0.84	101 (89.4)	50 (90.9)	0.76	151 (89.9)
Shock	51 (53.7)	25 (34)	**0.012**	56 (49.6)	20 (36.4)	0.11	76 (45.2)
Sepsis	16 (16,8)	5 (7)	0.05	17 (15)	4 (7.3)	0.15	21(12.5)
Trauma	0 (0)	2 (3)	0.10	0 (0)	2 (3.6)	**0.034**	2 (1.2)
Head injury	0 (0)	1 (1)	0.25	1 (0.9)	0 (0)	0.37	1 (0.6)
Cerebrovascular disease	2 (2.1)	7 (10)	**0.033**	4 (3.5)	5 (9.1)	0.13	9 (5.4)
Postoperative	27 (28.4)	16 (22)	0.34	30 (26.5)	13 (23.6)	0.68	43 (25.6)
Polypharmacy, *n* (%)	89 (93.7)	60 (82)	**0.020**	103 (91.2)	46 (83.6)	0.15	149 (88.7)
Body mass index, Median (25–75%)	25 (22–27)	27 (24–29)	**0.011**	26 (23–28)	26 (23–28)	0.39	26 (23–28)
Charlson comorbidity index, Median (25–75%)	8 (6–10)	5 (4–7)	**<0.001**	7 (5–9)	5 (4–8)	**<0.001**	7 (5–9)
SOFA Score, Median (25–75%)	8 (5–9)	6 (4–9)	**0.047**	7 (5–9)	6 (4–9)	**0.039**	7 (5–9)
Glasgow Coma Score, Median (25–75%)	9 (3–13)	11 (6–15)	**0.048**	9 (3–13)	11 (6–15)	0.058	9 (4–15)
Length of ICU stay, Median (25–75%)	8 (5–21)	9 (5–15)	0.63	8 (5–9)	9 (5–16)	0.75	8 (5–18)
Length of ICU stay, Median (25–75%)	8 (5–21)	9 (5–15)	0.63	8 (5–9)	9 (5–16)	0.75	8 (5–18)
Mechanical ventilation, *n* (%)	88 (92.6)	64 (87.7)	0.278	101 (89.4)	51 (92.7)	0.488	152 (90.5)
Vasopressors/vazoactive drugs, *n* (%)	78 (82.1)	39 (53.4)	<0.001	89 (78.8)	28 (50.9)	<0.001	117 (69.6)
Renal replacement therapy, *n* (%)	20 (21.1)	14 (19.2)	0.764	25 (22.1)	9 (16.4)	0.383	34 (20.2)
Tracheostomy, *n* (%)	22 (23.2)	19 (26.0)	0.668	24 (21.2)	17 (30.9)	0.171	41 (24.4)
Length of ICU stay, Median (25–75%)	8 (5–21)	9 (5–15)	0.63	8 (5–9)	9 (5–16)	0.75	8 (5–18)
28-day mortality, *n* (%)	34 (35.8)	12 (16)	**0.005**	38 (33.6)	8 (14.5)	**0.009**	46 (27.4)

Bold values are statistically significant (*p* < 0.05). ICU, intensive care unit; NRS-2002, Nutritional Risk Screening 2002; APACHE II, Acute Physiology and Chronic Health Evaluation II.

The median Charlson Comorbidity Index was 7 (IQR 5–9), APACHE II score 23 (18–30), and GCS 9 (IQR 4–15). The median BMI was 26 (23–28) and NRS-2002 4 (3–5). On ICU admission, 95 patients (56.5%) were classified as frail according to the FRAIL scale, and 113 (67.3%) according to the CFS. Mechanical ventilation was used in 152 patients (90.5%), vasopressors/vazoactive drugs in 117 (69.6%), renal replacement therapy in 34 (20.2%), and tracheostomy in 41 (24.4%).

**Table:** 

Intervention	*n* (%)	Median duration among users, days (IQR)
Mechanical ventilation	152 (90.5)	7.5 (3.0–16.25)
Vasopressors/vazoactive drugs	117 (69.6)	5.0 (2.0–8.0)
Renal replacement therapy	34 (20.2)	3.0 (2.0–6.0)
Tracheostomy	41 (24.4)	14.0 (6.0–24.0)

FRAIL-defined frail patients were older and had higher Charlson Comorbidity Index, APACHE II, SOFA, polypharmacy, and NRS-2002 values, as well as lower BMI and GCS values, than non-frail individuals. CFS-defined frail patients were older and had higher Charlson Comorbidity Index, APACHE II, and SOFA scores; however, the NRS-2002 difference was not statistically significant for CFS-defined frailty (Table 1). Additional comparisons of ICU interventions showed that vasopressor/vazoactive drug use was significantly higher in frail patients according to both the FRAIL scale (82.1% vs. 53.4%, *p* < 0.001) and the CFS (78.8% vs. 50.9%, *p* < 0.001), whereas mechanical ventilation, renal replacement therapy, and tracheostomy did not differ significantly between frail and non-frail patients on either scale (Table 1).

### 3.2 Outcomes

Overall, 46 patients (27.4%) died within 28 days of ICU admission, and the median ICU length of stay was 8 days (IQR 5–18). Patients classified as frail by the FRAIL scale had a higher 28-day mortality rate compared with non-frail patients (36% vs. 16%, *p* = 0.005; crude OR 2.83, 95% CI 1.34–6.00). Similarly, mortality was higher among frail patients as defined by the CFS (34% vs. 15%, *p* = 0.009; crude OR 2.98, 95% CI 1.27–6.97).

**Figure 2:**
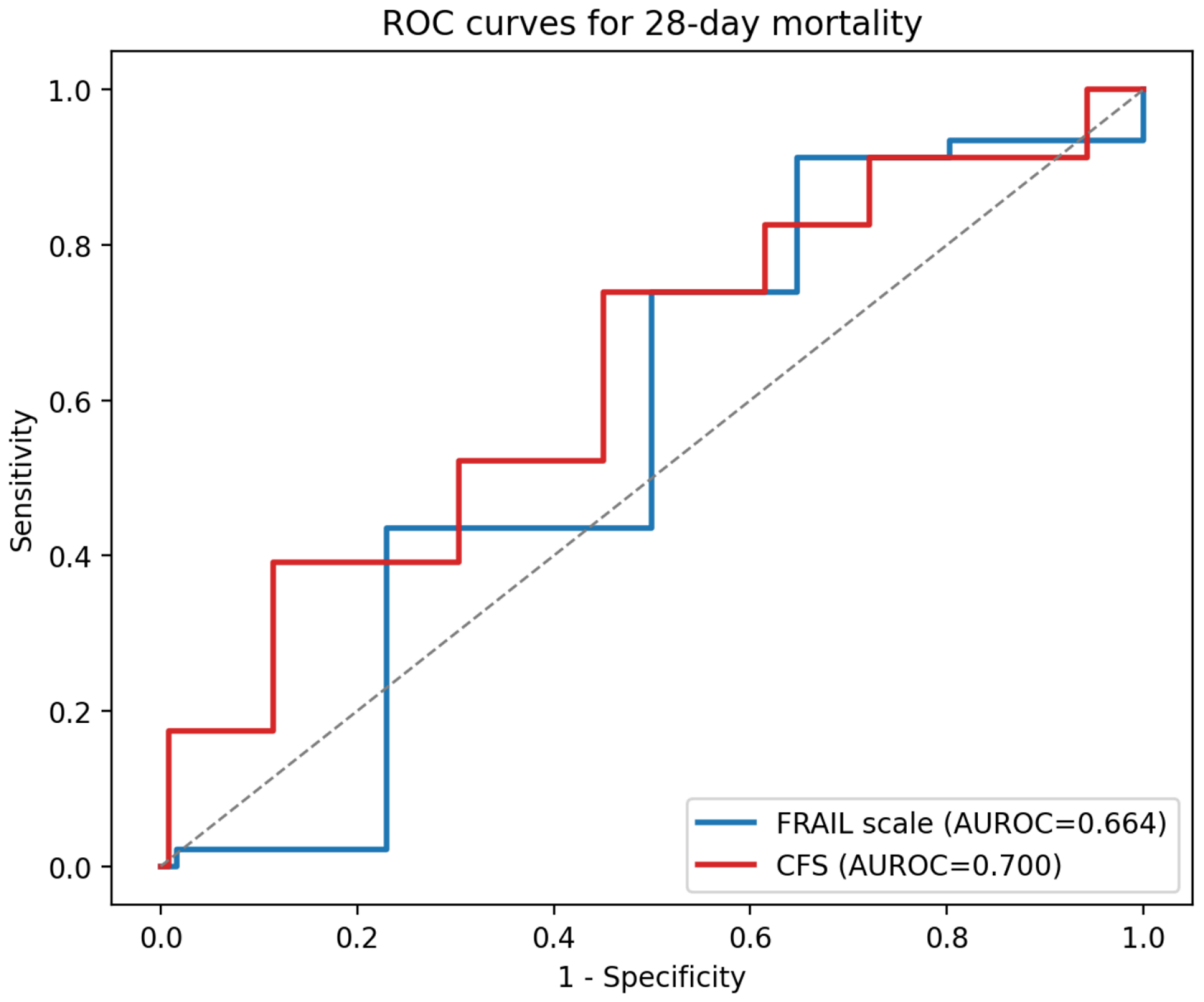
Receiver operating characteristic curves for FRAIL and CFS total scores predicting 28-day mortality.

**Figure 3:**
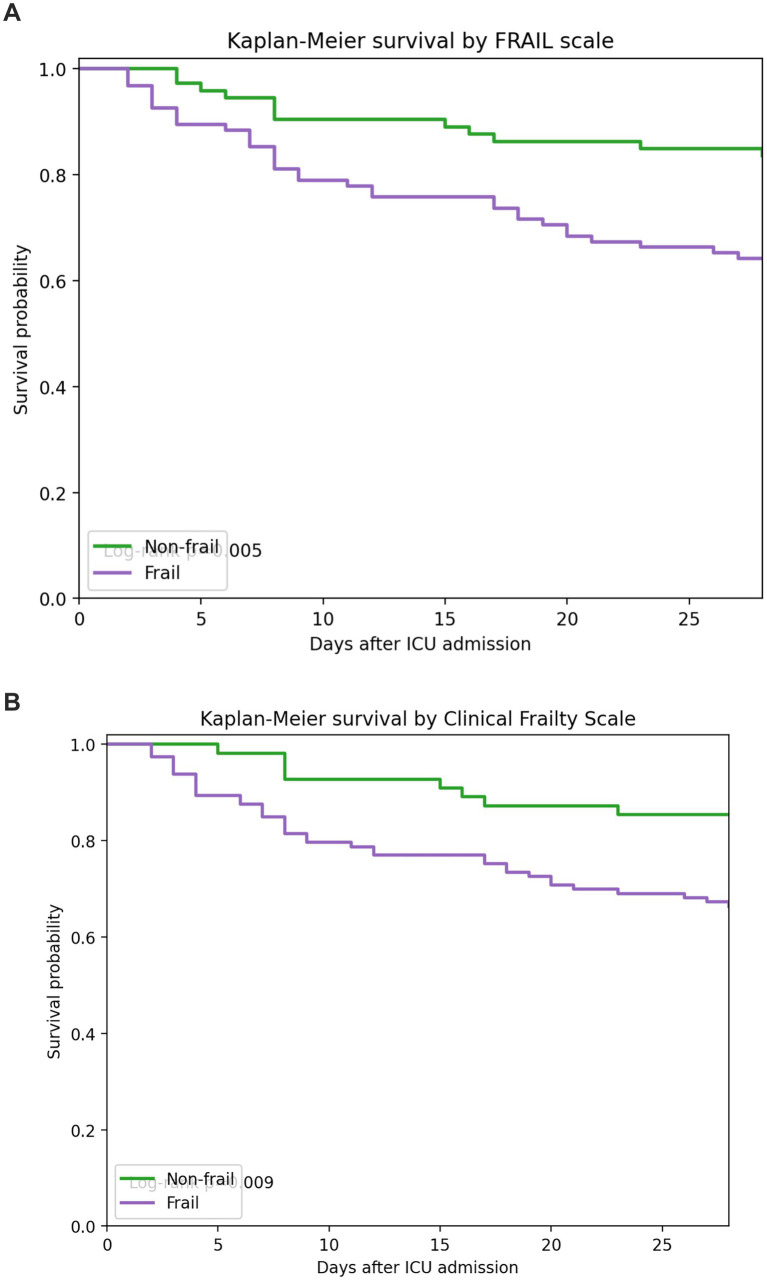
**(A)** Kaplan–Meier survival curves according to FRAIL-defined frailty (log-rank *p* = 0.005). **(B)** Kaplan–Meier survival curves according to CFS-defined frailty (log-rank *p* = 0.009).

AUROC analysis showed that total-score discrimination was 0.664 (95% CI 0.578–0.749) for the FRAIL scale and 0.700 (95% CI 0.611–0.789) for the CFS; this difference was not statistically significant by DeLong testing (*p* = 0.149). For dichotomized frailty status, AUROC values were 0.620 (95% CI 0.541–0.698) for the FRAIL scale and 0.606 (95% CI 0.535–0.676) for the CFS (DeLong *p* = 0.595; Figure 2). Kaplan–Meier analysis showed lower 28-day survival in frail patients according to both the FRAIL scale (log-rank *p* = 0.005) and the CFS (log-rank *p* = 0.009; Figure 3).

In multivariable logistic regression analysis, frailty defined by the FRAIL scale remained independently associated with 28-day mortality (adjusted OR 2.41, 95% CI 1.01–5.76; *p* = 0.048) after adjustment for age, BMI, Charlson Comorbidity Index, APACHE II score, GCS, NRS-2002, shock, cerebrovascular disease, and polypharmacy (Table 2). However, CFS-based frailty was not independently associated with mortality in the CCI-adjusted model (adjusted OR 2.26, 95% CI 0.91–5.63; *p* = 0.080; Table 3). In sensitivity analyses excluding CCI, FRAIL-defined frailty remained significant (OR 2.77, 95% CI 1.17–6.54; *p* = 0.020), and CFS-defined frailty also became significant (OR 2.59, 95% CI 1.05–6.38; *p* = 0.038).

**Table 2:** Association of the FRAIL scale with 28-day mortality (multivariable logistic regression).

Variable	*p*	OR	95% CI for OR	
			Lower	Upper
FRAIL	**0.048**	**2.412**	**1.010**	**5.761**
Shock	0.246	1.602	0.722	3.555
Cerebrovascular disease	0.994	0.993	0.172	5.743
Glasgow coma score	**0.040**	**0.904**	**0.822**	**0.995**
NRS-2002	0.231	1.235	0.874	1.743
Polypharmacy	0.235	0.363	0.068	1.929
Age	0.669	1.011	0.963	1.060
Body mass index	0.060	1.090	0.997	1.193
Charlson comorbidity index	0.089	1.131	0.982	1.303
APACHEII	0.949	0.998	0.944	1.055

Bold values are statistically significant (*p* < 0.05). ICU, intensive care unit; NRS-2002, Nutritional Risk Screening 2002; APACHE II, Acute Physiology and Chronic Health Evaluation II.

**Table 3:** Association of the Clinical Frailty Scale with 28-day mortality (multivariable logistic regression).

Variable	*p*	OR	95% CI for OR	
			Lower	Upper
CFS	0.080	2.259	0.907	5.627
Age	0.926	1.002	0.957	1.049
Charlson comorbidity index	**0.023**	**1.161**	**1.020**	**1.320**
APACHE II	0.267	1.025	0.981	1.071

Bold values are statistically significant (*p* < 0.05). ICU, intensive care unit; NRS-2002, Nutritional Risk Screening 2002; APACHE II, Acute Physiology and Chronic Health Evaluation II.

Agreement between the FRAIL and CFS measures was good (*κ* = 0.75, 95% CI 0.65–0.85; Table 4).

**Table 4:** Agreement between the FRAIL scale and Clinical Frailty Scale (Cohen’s kappa analysis).

FRAIL scale classification		Clinical Frailty Scale (CFS) classification		
		Frail	Not frail	Total
FRAIL	Frail	94 (83.2%)	1 (1.8%)	95 (56.5%)
	Not Frail	19 (16.8%)	54 (98.2%)	73 (43.5%)
	Total	113 (100%)	55 (100%)	168 (100%)

The agreement between CFS and FRAIL scale scores in geriatric patients hospitalized in the ICU was good (Kappa 0.75, 95% CI, 0.65–0.85, Table 4).

## 4 Discussion

This study demonstrates that frailty assessed at ICU admission is a significant prognostic factor for short-term mortality in older adults. Although both the CFS and FRAIL scales were associated with increased 28-day mortality, only the FRAIL scale remained an independent predictor in the primary CCI-adjusted model. However, AUROC and DeLong analyses did not demonstrate a statistically significant discrimination advantage for either instrument. These findings suggest that while both instruments capture elements of physiological vulnerability, the FRAIL scale may provide independent prognostic information in conservative adjusted modelling. To our knowledge, this is one of the first prospective studies to directly compare the FRAIL and Clinical Frailty Scale tools in a critically ill geriatric population, addressing an important gap in the current literature.

Frailty reflects diminished physiological and functional reserves, which limits an individual’s capacity to recover from acute stressors. The higher mortality observed among frail patients in our cohort aligns with previous studies demonstrating similar trends. Kalaiselvan et al. (7) reported a 1-month mortality rate of 49% in frail ICU patients compared with 29% in non-frail individuals, while López Cuenca et al. (8) and de Geer et al. (9) also showed increased 30-day mortality associated with frailty. The latter study further found that combining the CFS with the Simplified Acute Physiology Score III improved predictive accuracy (9). The FORECAST multicentre cohort also supports the prognostic and recovery-related importance of frailty assessment in critically ill patients (22). These consistent findings across studies reinforce the notion that frailty represents a distinct biological risk factor beyond chronological age or disease severity.

In the present study, multivariable logistic regression revealed that frailty defined by the FRAIL scale remained independently associated with 28-day mortality after adjusting for age, BMI, Charlson Comorbidity Index, APACHE II score, GCS, NRS-2002, shock, cerebrovascular disease, and polypharmacy. However, frailty defined by the CFS did not retain statistical significance in this conservative CCI-adjusted model. Because comorbidity may be considered both a confounder and a component of frailty biology, sensitivity analyses excluding CCI were performed; in these analyses, both FRAIL-defined and CFS-defined frailty were associated with mortality. While the CFS is a well-established and practical bedside tool, its reliance on functional dependency may overlap with comorbidity and acute illness severity in older ICU patients. This difference may explain why the FRAIL scale demonstrated independent prognostic value in the primary adjusted model, although AUROC comparison did not support a statistically significant discrimination advantage.

Several classification systems have been developed to define frailty, but their prognostic validity can vary depending on the clinical setting (15, 23). The CFS, a nine-point ordinal scale, has been widely used in both acute and chronic care environments. In contrast, the FRAIL scale provides a simpler, symptom-based framework that encompasses physical performance, nutritional status, and illness burden—all factors that may directly influence outcomes in critical illness. Its multidimensional approach may therefore make it more suitable for assessing older adults at ICU admission.

By demonstrating that the FRAIL scale retained independent predictive value for short-term mortality, our findings address a key gap in the literature and highlight the need to consider alternative frailty assessment tools in intensive care practice. Future studies with larger sample sizes and multicentre designs are warranted to validate these findings and to determine whether routine use of the FRAIL scale can improve prognostic stratification and guide individualized care planning in the ICU.

The CFS has gained widespread attention for its simplicity and validation across multiple medical settings (4, 10). In intensive care, it remains the most extensively studied frailty tool and was therefore included in our analysis. However, unlike findings from large-scale ICU studies confirming the prognostic value and reliability of the CFS (10, 22, 23), our primary CCI-adjusted model indicated limited independent prognostic performance after adjustment for comorbidities and illness severity. A recent Acta Medica study of octogenarian ICU patients also supports the clinical relevance of CFS-based frailty assessment, while showing that survival patterns across frailty categories may vary by population and design (24). This discrepancy may be attributed to the substantial comorbidity burden and critical illness severity commonly observed in elderly ICU patients, which can mask the independent effect of frailty as measured by the CFS.

In contrast, the FRAIL scale demonstrated independent predictive value for 28-day mortality in the primary adjusted model. Although less studied in intensive care populations, its multidimensional nature—capturing fatigue, mobility, illness burden, and weight loss—may explain its prognostic capacity. Given its simplicity and feasibility at ICU admission, the FRAIL scale could serve as a practical tool for early identification of high-risk older patients. De Biasio et al. (11) similarly suggested that frailty instruments like the FRAIL scale enable clinicians to recognize vulnerable patients earlier, facilitating targeted interventions and improved outcomes. Routine implementation of such tools may also enhance long-term prognostic stratification in geriatric ICU care. Importantly, the agreement between the CFS and FRAIL scores in our study was good, supporting the concurrent use of both instruments for a more comprehensive frailty assessment in critically ill older adults.

More than half of our cohort were men, consistent with previous studies (25). Although frailty has long been a cornerstone of geriatric medicine, its role as a determinant of prognosis in critically ill patients has only recently been recognized. Our findings align with those of Bruno et al. (23), who reported that the CFS predicted mortality among older but not younger ICU patients. Consistent with prior research, frail patients in our cohort were older, had higher Charlson Comorbidity, APACHE II, and SOFA scores, and were more likely to exhibit polypharmacy. They also had lower BMI and GCS values, likely reflecting diminished physiological and cognitive reserves characteristic of frailty (26).

Most patients were admitted from home, and frailty prevalence did not differ significantly by living environment on either scale. This contrasts with Akdag et al. (27), who found higher FRAIL-defined frailty among community-dwelling older adults compared with nursing home residents. The difference may relate to the predominance of home-dwelling elderly in our cohort, who might have limited access to regular medical follow-up, leading to unrecognized frailty that only becomes apparent during acute critical illness.

The majority of patients in our study were admitted to the ICU with respiratory failure, consistent with prior reports (25, 28). Older adults are particularly susceptible to respiratory failure due to age-related physiological changes that diminish cardiopulmonary reserves and increase the need for mechanical ventilation support (27). Interestingly, in our cohort, frailty as measured by the FRAIL scale was significantly higher among patients admitted with shock. This may be explained by the greater systemic stress and multisystem involvement inherent to shock, which disproportionately affect individuals with limited physiological resilience. In addition, frail patients often have reduced cardiovascular reserves, predisposing them to hemodynamic instability and a higher likelihood of developing shock rather than isolated respiratory failure (13).

Frailty has been consistently associated with increased mortality, although the underlying mechanisms remain incompletely understood. Recent evidence suggests that lifestyle factors, social support, psychological health, cognitive function, and physical resilience may mediate this relationship (29). Identifying these mediators could help clarify the biological and behavioural pathways linking frailty to adverse outcomes and support targeted interventions to improve survival in older adults.

### 4.1 Limitations

This study has several limitations. First, its single-centre design may restrict the generalisability of findings to other ICU settings. Second, while the prospective design allowed for accurate data collection, the observational nature of the study limits causal inference. Third, although the CFS and FRAIL scales are validated instruments, they may not fully capture the dynamic and multidimensional nature of frailty, including its temporal changes during critical illness. Fourth, exclusion of patients discharged alive or dying within 24 h may have influenced the apparent performance of the two frailty instruments differently; very early deaths may be driven predominantly by acute catastrophic illness, whereas very early discharges may include lower-risk admissions. Finally, although multivariable logistic regression was used to adjust for potential confounders, residual confounding cannot be entirely excluded. Future multicentre longitudinal studies using comprehensive frailty assessments are warranted to validate these results and further elucidate the prognostic role of frailty in critically ill older adults.

## 5 Conclusion

Frailty at ICU admission is an important prognostic marker for short-term mortality in older adults. In this prospective cohort study, the FRAIL scale remained an independent predictor after adjustment for illness severity and comorbidities, while AUROC comparison did not demonstrate a statistically significant discrimination advantage over the CFS. These findings suggest that the FRAIL scale may offer practical and clinically relevant value for mortality risk stratification in critically ill geriatric patients, but claims of comparative advantage should be interpreted cautiously. Routine frailty assessment at ICU admission may improve early risk identification and support individualized care planning.

## Data Availability

The raw data supporting the conclusions of this article will be made available by the authors, without undue reservation.
